# Segmental hepatic necrosis following embolization for delayed hemorrhage after laparoscopic pancreaticoduodenectomy: a case report

**DOI:** 10.3389/fmed.2026.1787056

**Published:** 2026-03-04

**Authors:** Jinglong Sun, Zhenhua Li, Jianguo Jia, Yuzhen Gong, Zhongying Li, Zhirun Dong, Xi Wang, Xiangming Ma

**Affiliations:** 1 Department of Hepatobiliary Surgery, Kailuan General Hospital Affiliated North China University of Science and Technology, Tangshan, China; 2 Department of Orthopedics, The People’s Hospital of Tengxian, Wuzhou, China; 3 Department of Traditional Chinese Medicine, The Second Affiliated Hospital of Xingtai Medical College, Xingtai, China

**Keywords:** case report, delayed hemorrhage, embolization, hepatic necrosis, pancreaticoduodenectomy

## Abstract

Postpancreaticoduodenectomy hemorrhage (PPH) is a serious complication of laparoscopic pancreaticoduodenectomy (LPD); however, hepatic lobe necrosis secondary to transarterial embolization for this condition is exceedingly rare. To our knowledge, this complication has been sparsely documented, with previous reports predominantly focusing on treatment modality selection for PPH rather than on post-embolization hepatic necrosis. We report a case of late intra-abdominal hemorrhage after LPD, which was treated with common hepatic artery embolization under digital subtraction angiography (DSA). Forty days after embolization, necrotic tissue was spontaneously discharged from the laparotomy wound, and histopathology revealed devitalized liver parenchyma. The patient recovered well with conservative wound care, drainage, and irrigation. Hepatic necrosis following embolization for PPH may result from the left lobe’s paucity of collaterals, which leads to insufficient perfusion and renders it more vulnerable to ischemic injury. This case underscores the critical importance of preoperative imaging and surgical planning in LPD. Preoperative imaging enables precise delineation of hepatic vascular anatomy, facilitating comprehensive surgical planning to maximize preservation of major vessels and enabling informed decision-making even when managing PPH.

## Introduction

Over the past three decades, perioperative mortality after LPD has fallen dramatically. This improvement reflects refinements in surgical technique, enhanced perioperative care, and advances in critical-care medicine. Nevertheless, postoperative morbidity remains unchanged following LPD (1–3). Postpancreaticoduodenectomy hemorrhage (PPH) is uncommon; however, it carries a mortality rate as high as 16–50% (4).

In the treatment of PPH, several studies suggest that early PPH is best managed by prompt relaparotomy. However, the optimal strategy for delayed PPH remains controversial. The general assumption is that minimally invasive endovascular therapy currently offers the best available treatment, whether through embolization or covered stents (5). Severe delayed postoperative hemorrhage requires immediate surgical hemostasis in two scenarios: 1. when it is refractory to interventional therapy, or 2. when associated with hemodynamic instability (6).

Moreover, delayed PPH is often precipitated by postoperative pancreatic fistula (POPF) with secondary intraabdominal abscess. Therefore, thorough drainage of the fistula and any infected collections is equally critical (7). Previous investigations of PPH following LPD have predominantly addressed treatment algorithm selection and technical aspects of embolization; by contrast, hepatic lobe necrosis as a complication of transarterial embolization has not been previously documented, representing a significant knowledge gap.

This case describes a patient with delayed hemorrhage after LPD who was successfully rescued after reoperation and interventional embolization, but shortly thereafter developed partial necrosis of the left hepatic lobe. This experience provides valuable insights into the management of embolization-induced hepatic necrosis. It also underscores that preoperative imaging assessment of hepatic vascular variants is essential. Specifically, such evaluation minimizes procedure related morbidity and foster advancement of LPD.

## Case description

A 64-year-old man was referred for newly detected bile-duct dilatation. Although CA19-9, total bilirubin (TBIL) and direct bilirubin (DBIL) were normal, markedly elevated *γ*-glutamyl transferase (γ-GT) along with contrast-enhanced CT and MRI findings indicated a neoplastic lesion in the distal bile duct. After obtaining written informed consent, laparoscopic pancreaticoduodenectomy was performed on 20 February 2025. The operation proceeded uneventfully with an estimated blood loss of less than 50 mL. Final pathology report confirmed: High-grade villous adenoma of the distal common bile duct with focal adenocarcinomatous transformation; neoplasm limited to the mucosa without muscular layer invasion, perineural invasion, or pancreatic parenchymal involvement.

On postoperative day (POD) 3, based on the ISGPS definition and grading of POPF, the patient was classified as grade C (8). Daily irrigation with normal saline was administered to dilute the drain effluent and maintain catheter patency, suppression of pancreatic exocrine secretion and antibiotic therapy. The POPF had improved since the last evaluation. On March 19, apart from cloudy grey fluid still draining from the preanastomotic drain, all other tubes had been removed; the patient was discharged with repeated instructions to maintain unobstructed drainage.

**Figure 1:**
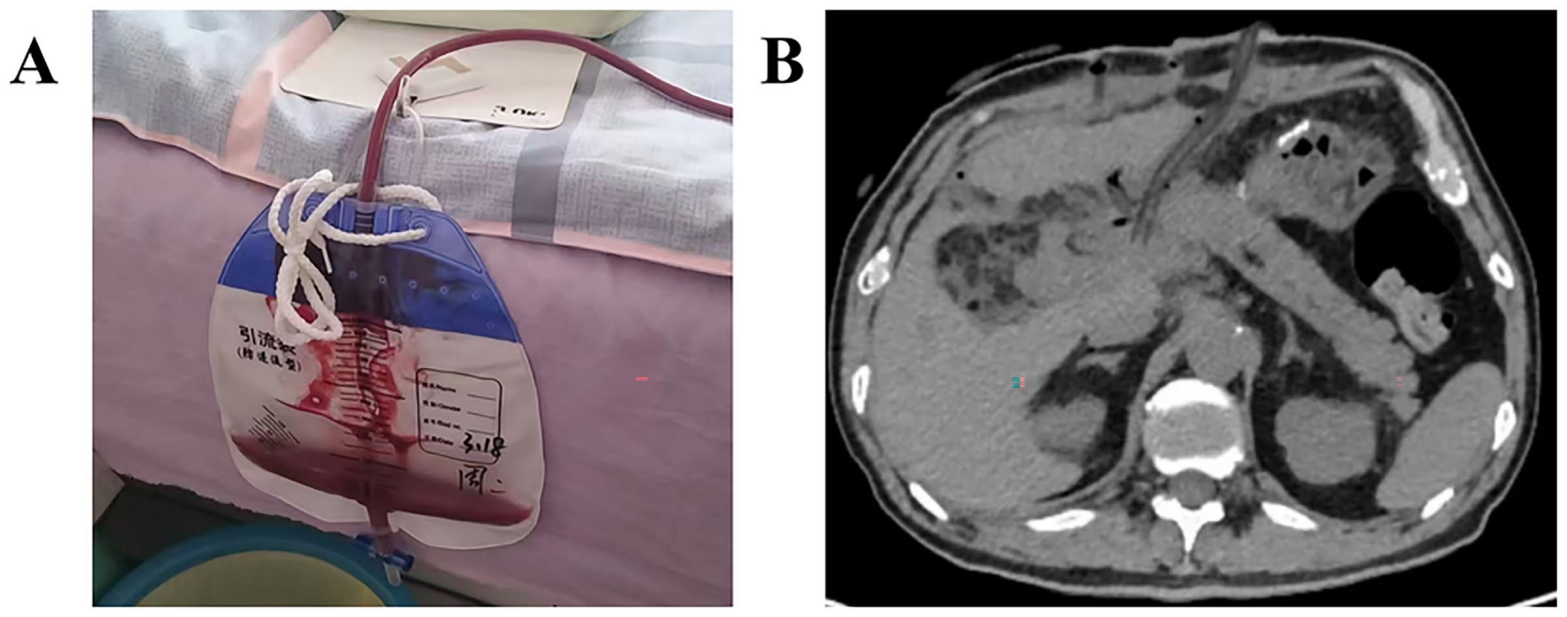
**(A)** The patient remained hemodynamically stable; a sizeable accumulation of bloody fluid in the drainage bag raised our suspicion of sentinel bleeding. **(B)** CT showing drainage tubes placed for POPF management to prevent further vascular erosion after emergency laparotomy for initial PPH.

On POD 32, the patient experienced sudden abdominal pain and intermittent bloody drainage totaling 75 mL, raising concern for sentinel bleeding (Figure 1A); he was instructed to return to the hospital immediately. Twenty hours later, the patient developed massive intra-abdominal bleeding of approximately 1,000 mL (including irrigation fluid). Emergency laparotomy revealed that a POPF had eroded the Gastroduodenal Artery (GDA) stump, causing the bio-clip and silk ligature to dislodge. Bleeding was effectively controlled with Prolene sutures, two drains were placed, and continuous lavage drainage was instituted post-operatively to prevent further erosion with POPF (Figure 1B).

**Figure 2:**
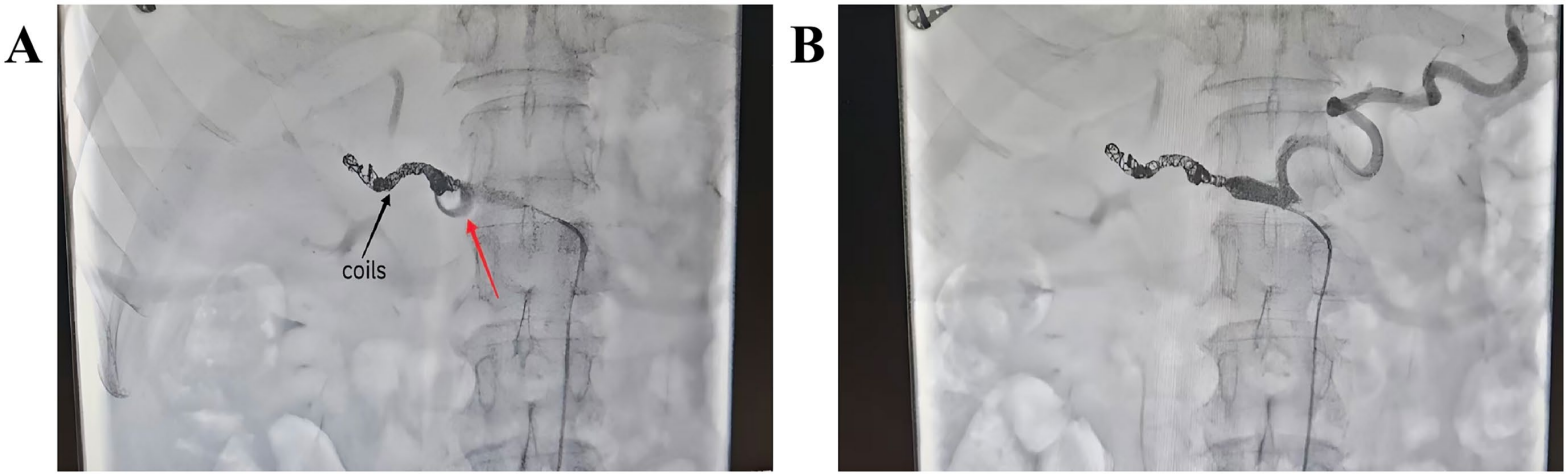
Digital subtraction angiography (DSA) showing: **(A)** Transient GDA stump extravasation (red arrow) and common hepatic artery coil embolization (black arrow); **(B)** Complete arterial embolization without contrast extravasation.

Unfortunately, on POD 39, the patient experienced another severe intraperitoneal hemorrhage. Following multidisciplinary review, digital subtraction angiography (DSA) with embolization for hemostasis was performed. The bleeding site was again identified at the GDA stump. Before the embolization procedure, a comprehensive analysis was conducted. It revealed that the patient had a history of recurrent PPH with significant bleeding volume, which necessitated prompt and effective hemostasis to avoid the risks of reoperation. Furthermore, the DSA demonstrated that the GDA stump was too short to allow effective embolization for hemostasis. Therefore, the common hepatic artery was embolized, achieving secure hemostasis (Figure 2).

**Figure 3:**
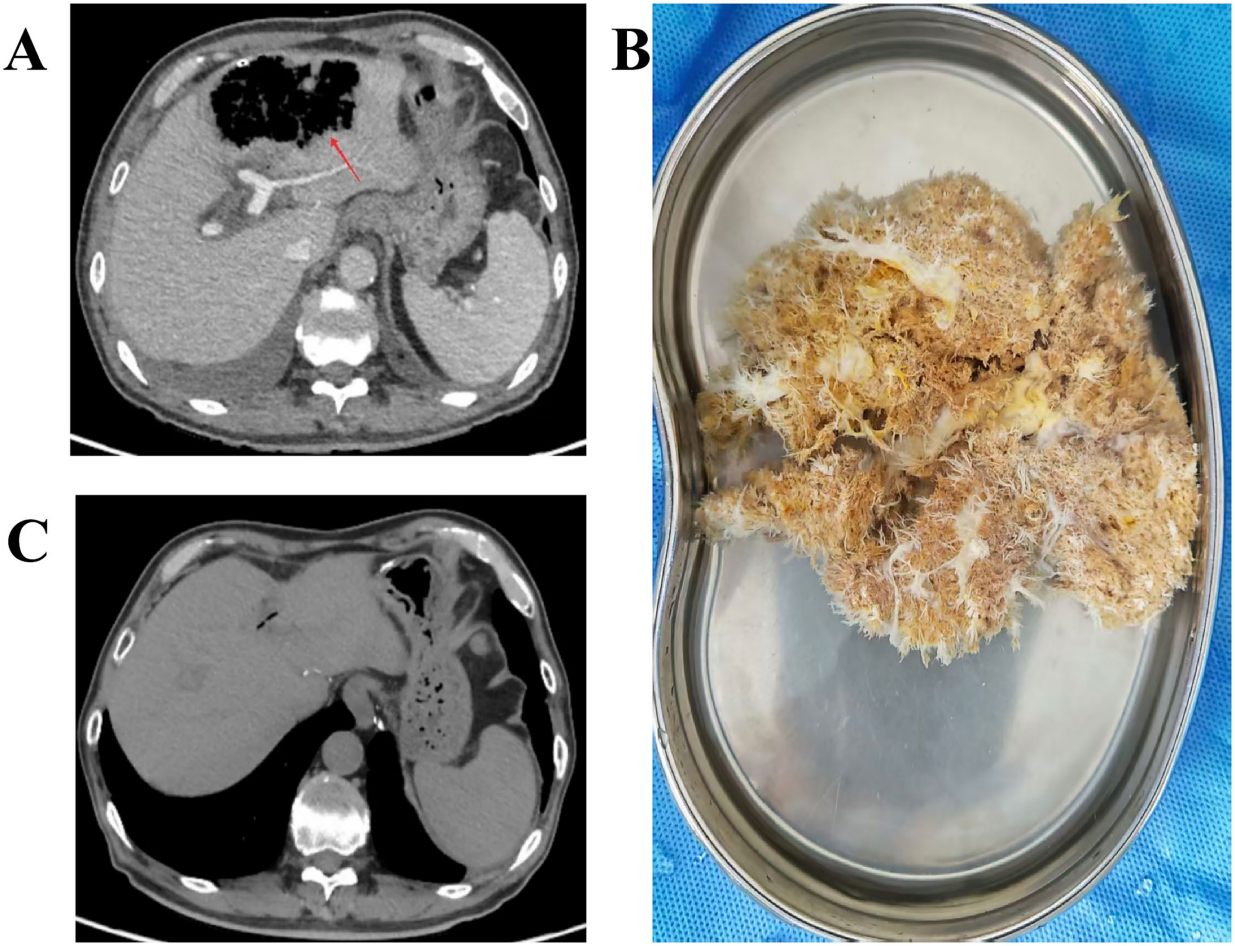
**(A)** CT showing focal intrahepatic gas accumulation in segments II and III following common hepatic artery embolization (red arrow). **(B)** Necrotic liver tissue extruded from the laparotomy wound. **(C)** CT showing regeneration of liver tissue within the previous defect of the left hepatic lobe at 4 months after LPD.

On post-embolization day 1, the patient experienced episodic chills and fever, with peak temperatures up to 39 °C. Blood cultures grew *Klebsiella pneumoniae;* therefore, antimicrobial therapy was adjusted, and traditional Chinese medicine (heat-clearing and detoxifying therapy) was added. A follow up CT revealed focal intrahepatic gas accumulation in segments II and III (Figure 3A); Given the patient’s symptoms and elevated liver enzymes (aspartate aminotransferase (AST) 532 U/L, alanine aminotransferase (ALT) 331 U/L; pre-embolization: AST 20 U/L, ALT 25 U/L), hepatic necrosis was suspected. To prevent further worsening of the infection, an ultrasound-guided percutaneous liver drainage of the abnormal area in the left lateral hepatic lobe was performed. In addition, the peritoneal cavity was continuously irrigated with normal saline. After approximately 40 days of continuous dressing changes, lavage, and drainage, the POPF resolved completely.

On May 11, dressing change revealed fresh dehiscence of the previously healed laparotomy wound, with necrotic tissue protruding from the incisional depths, which was promptly removed. After irrigation, the specimen appeared to be devitalized liver parenchyma (Figure 3B). Histopathology revealed necrotic liver tissue, corroborating the previous CT finding of a focal lesion in the left hepatic lobe. Following removal of the necrotic tissue, the patient reported no discomfort and remained hemodynamically stable. Daily peritoneal lavage with normal saline was continued. In subsequent care the disrupted wound healed completely, with no drainage or odor. Repeat laboratory studies were unremarkable. Four months later, Outpatient CT showed regeneration of liver tissue within the previous defect of the left hepatic lobe (Figure 3C).

## Discussion

We report a 64-year-old man who underwent pancreaticoduodenectomy. Postoperatively he developed recurrent bleeding from the GDA stump, precipitated by POPF erosion. Hemostasis was finally secured after successive interventions: initial laparotomy, followed by DSA with embolization. 40 days after embolization a previously healed laparotomy wound dehisced during routine dressing, and necrotic liver tissue extruded spontaneously. Previous studies have reported hepatic infarction in approximately 30% of patients undergoing embolization for bleeding after pancreaticobiliary surgery (9, 10). These studies indicate that hepatic infarction or abscess following hepatic artery embolization for post-pancreaticobiliary bleeding is not uncommon but rather an anticipated complication that demands proactive prevention.

A previous case report documented cases of hepatic abscess formation following embolization for PPH after LPD, with subsequent resolution (11). Qingyun et al. (12) and Lifeng et al. (13) among others described a patient with severe delayed hemorrhage following LPD who underwent successful hemostasis via common hepatic artery embolization; however, no post-embolization hepatic necrosis or related complications were reported. No prior case of hepatic necrosis discharged via the laparotomy wound after embolization had been documented.

In PPH, the GDA stump is the most frequent bleeding site, followed by the hepatic artery and its branches (14). For GDA stump bleeding, there are two interventional embolization methods for hemostasis: embolization of the hepatic artery proximal and distal to GDA stump and selective embolization of the GDA stump. Previous studies have demonstrated that embolization of the hepatic artery proximal and distal to GDA stump achieves optimal hemostasis (15). Other studies have also shown that selective embolization of the GDA stump achieves hemostatic outcomes comparable to the former technique (16). The liver receives blood from two sources: the hepatic artery (25%) and the portal vein (75%). While the hepatic artery accounts for only a quarter of the inflow, it supplies about half of the liver’s oxygen (17). Thus, hepatic arterial perfusion is essential; common hepatic artery embolization significantly impairs hepatocyte viability and function in patients with insufficient collateral circulation.

Interestingly, most of these events occurred predominantly in the left lobe. This case report is no exception. Literature indicates that after hepatic artery embolization the liver develops collateral circulation for compensation; however, collateral arterial flow occurs significantly more often in the right liver than in the left. In other words, the left lobe forms fewer collaterals, resulting in insufficient perfusion and rendering it more vulnerable to ischemic injury (18).

Preoperative vascular imaging plays a critical role in LPD. Computed Tomography Angiography (CTA) or Magnetic Resonance Angiography (MRA) is particularly important in patients with prior surgical history or anatomical variations. It enables both evaluation of tumor vascular invasion and detailed visualization of critical anatomy and collateral pathways, facilitating improved surgical results (19). Hepatic arterial variants are common, occurring in up to 45% of individuals, and present substantial challenges in minimally invasive surgery (20). For example, preoperative imaging should be performed to identify any replaced or accessory left hepatic artery originating from the left gastric artery, and this vessel should be preserved whenever clinically feasible (21). Moreover, the right inferior phrenic artery constitutes the most frequent collateral pathway.

Endovascular treatment has increasingly become the preferred approach for PPH following LPD, offering the advantages of avoiding laparotomy while achieving significant hemostatic efficacy (22). These include embolization (superselective and non-selective) and covered stents. Based on the lesion type, local hemodynamics, and feeding artery anatomy, various embolic agents can be employed to achieve successful endovascular hemostasis. Superselective embolization (e.g., GDA stump embolization) and covered stents enable effective bleeding control with preserved hepatic perfusion, reducing ischemic risk (23). Nevertheless, these approaches have certain limitations. Superselective embolization is limited by the requirement for sufficient GDA stump length and is associated with a higher rebleeding rate than common hepatic artery embolization (16). Covered stents are constrained by small or tortuous vessel anatomy and necessitate long-term dual antiplatelet therapy (24). In the present case, the patient presented with recurrent PPH and had already undergone repeat laparotomy for bleeding control. Given the inadequate length of the GDA stump, common hepatic artery embolization was elected. The main complication of this approach is insufficient hepatic perfusion. This case illustrates the complex management of postoperative complications following LPD and the rare spontaneous expulsion of necrotic hepatic tissue after embolization. This case underscores the risk of hepatic necrosis following embolization for PPH.

## Conclusion

This case presents a rare but clinically significant complication following common hepatic artery embolization for delayed PPH after LPD: spontaneous extrusion of necrotic liver tissue via the laparotomy wound. The left hepatic lobe, owing to its limited collateral supply, appears particularly vulnerable to ischemic injury after common hepatic artery embolization. This event underscores the critical importance of preoperative imaging in LPD to identify vascular variants and guide surgical planning. Furthermore, when endovascular treatment hemostasis is required, the choice of technique—whether superselective embolization, common hepatic artery embolization, or covered stents—should be carefully weighed against the patient’s anatomy and collateral reserve. These features underscore the importance of preoperative vascular assessment in LPD, as well as the need for prompt management of post-embolization hepatic ischemic necrosis.

## Data Availability

The original contributions presented in the study are included in the article/supplementary material, further inquiries can be directed to the corresponding authors.
